# Assessment of Early Cognitive Impairment in Patients with Clinically Isolated Syndromes and Multiple Sclerosis

**DOI:** 10.1155/2014/637694

**Published:** 2014-04-14

**Authors:** Leyla Baysal Kıraç, Özgül Ekmekçi, Nur Yüceyar, Ayşe Sağduyu Kocaman

**Affiliations:** ^1^Neurology Department, School of Medicine, Ege University, 35100 Bornova, İzmir, Turkey; ^2^Neurology Department, Acibadem University School of Medicine, Turkey

## Abstract

*Objective*. The aim of our study was to investigate the frequency and pattern of cognitive impairment in patients with clinically isolated syndromes and definite diagnosis of multiple sclerosis within the last 2 years. *Methods*. We assessed the cognitive status of 46 patients aged 18–49 years with clinically isolated syndromes or definite diagnosis of multiple sclerosis who have onset of their symptoms within the last 2 years. Patients were matched with 40 healthy participants for age, sex, and educational level. Neuropsychological assessment was performed by stroop test, paced auditory serial addition test (PASAT), controlled oral word association test (COWAT), clock drawing test, trail making test (TMT), faces symbol test (FST). Hamilton Depression Scale and Modified Fatigue Impact Scale were used to quantify the severity of any depression and fatigue the subjects might suffer. *Results*. 19.6% of early MS/CIS group failed at 4 and more tests and had significant cognitive impairment focused on attention, executive functions, memory, and learning. No significant relationship was found between cognitive impairment and disability and fatigue scores. *Discussion*. Cognitive impairment can be present from the earliest stage of multiple sclerosis. It should be considered among the main manifestations of MS even in the earliest stages of the disease.

## 1. Introduction

Cognitive dysfunction is increasingly recognized in early phase of multiple sclerosis (MS) and clinically isolated syndrome (CIS) patients [1–7]. MS related cognitive dysfunction is characterized by involvement of recent memory, attention, information processing speed, and executive dysfunction. MS patients with even mild cognitive deficits may experience greater difficulties at work and in social contact and daily activities, irrespective of physical handicap. Since cognitive deficits are not included in the definition of benign MS (EDSS Scores < 3 > 10 years after onset), the prevalence of benign disease may have been overestimated [8]. Early cognitive impairment in MS may predict disability outcome several years later [9, 10].

In this study we aimed to evaluate the frequency and pattern of cognitive impairment in patients with CIS and early phase of multiple sclerosis. We assessed relationship of cognitive impairment with the physical disability and fatigue. Furthermore we were interested in detecting a screening instrument that is simple, sensitive, and well tolerated by patients.

## 2. Methods

### 2.1. Subjects

Subjects aged between 18 and 50 with CIS or definite diagnosis of RRMS who have onset of their symptoms within the last 2 years were recruited from our multiple sclerosis outpatient department. CIS/MS subjects underwent a neurological examination that included a complete history and determination of current disability using the Expanded Disability Status Scale (EDSS) and neuropsychological evaluation. MRI of the patients was evaluated retrospectively. All RRMS patients fulfilled the MRI criteria which demonstrate dissemination of lesions in time and in space.

### 2.2. Clinical and Neuropsychological Assessment

The volunteers with major psychiatric illness, other neurological disease, learning disability, drug or alcohol abuse, and severe physical disability interfering tests were excluded from the study. To screen alcohol dependence and substance abuse we questioned the participants according to DSM-IV substance abuse and alcohol dependence criteria. We did not include the participants who had a drug abuse or alcohol dependence problem at some time in their life. All evaluations were performed at least 30 days after the end of a relapse. All patients fulfilled the 2005 Mc Donald's diagnostic criteria. All participants gave informed consent to participate in this study, which was approved by the Ethics Committee of the Hospital.

To assess cognition we created a battery with neuropsychological tests most of which have reliability and validity studies for Turkish patients lasting about 1.5 hour. We tried to evaluate several cognitive domains and chose the tests among the ones which were frequently used for evaluation of neurological disorders in Turkey. Hamilton Depression Status Scale was used to quantify the severity of depression. Participants with moderate and severe depression (who have scores over 16) were excluded from the study. To assess fatigue severity of the patients Modified Fatigue Impact Scale (MFIS) with physical, cognitive, and psychosocial subscales was used. The neuropsychological tests were considered to have failed when the results were outside the normal range (means ±2 SDs obtained by the healthy control group). Neuropsychological tests included the following.

*(A) Paced Auditory Serial Addition Test (PASAT)*. PASAT is a neuropsychological test used to assess auditory information processing speed, sustained attention, and calculation ability. The PASAT is presented on compact disk (CD) to control the rate of stimulus presentation. Single digits are presented either every 3 seconds (trial 1) or every 2 seconds (trial 2), and the patient must add each new digit to the one immediately prior to it. The test score is the total number of correct sums given (out of 60 possible) in each trial. The PASAT has regularly been reported to be a sensitive measure of cognitive impairment, and individuals with MS frequently perform more poorly than controls [11].

*(B) Faces Symbol Test (FST)*. FST is a culture free short screening instrument that measures attention, working memory and information processing speed. Participants first match 9 different faces with 9 different line symbols. Then match 67 faces with the right symbols. Total time to complete the test is calculated [12].

*(C) Rey Auditory and Verbal Learning Test (RAVLT).* RAVLT is used to interpret short term auditory verbal memory, learning, and retrieval. Participants are given a list of 15 unrelated words repeated over ten different trials and are asked to repeat. Then other tests were administered. After 40 minute delay the participants are asked to recall the original list of 15 words.

*(D) Stroop Test*. Stroop test measures selective attention, cognitive flexibility, and processing speed and is used in the evaluation of executive functions. When the name of a color is printed in a color not denoted by the name (e.g., the word “red” printed in blue ink instead of red ink), naming the color of the word takes longer and is more prone to errors. This is called Stroop effect. The patients did not have any color detection ability (the patients who suffered from optic neuritis were evaluated with “Ishihara cards” to screen for color vision detection ability before the test).

*(E) Trail making Test (TMT).* TMT measures visual attention, cognitive flexibility, and executive functions. The task requires a subject to “connect-the-dots” of 25 consecutive targets on a sheet of paper as fast as possible. There are two parts to the test: A, in which the targets are all numbers (1, 2, 3, etc.) and the test taker needs to connect them in sequential order, and B, in which the subject alternates between numbers and letters (1, A, 2, B, etc.).

*(F) Verbal Fluency Tests*. Controlled Oral Word Association Test (KAS in Turkish version for FAS was used) requires to say as many words as possible beginning with a designed letter within 60 seconds. Scores are the sum of all admissible words across the three trials. Semantic fluency was examined by asking the participants to name as many different animals as they could within 60 seconds. Verbal fluency tests evaluate attention, executive functions, verbal fluency, and retrieval.

*(G) Clock Drawing Test*. Clock drawing test is a quick screening test to assess abstract thinking, visuospatial organization, and executive functions. All subjects were given a sheet of paper with a predrawn circle on it. Subjects were then asked to “put the numbers on the clock and set the time at 10 after 11.” Scoring of the CDT was based on the method used by Shulman et al. [13]. In brief, the classification of CDT errors is as follows: 1 = perfect; 2 = minor visuospatial errors; 3 = inaccurate representation; 4 = moderate visuospatial disorganization; 5 = severe level of disorganization; 6 = no reasonable presentation of a clock.

### 2.3. Data Analysis

All statistical analyses were performed using SPSS for Windows version 15.0. The significance level was set at 0.05. The correlations between the demographic parameters and the cognitive subgroups were tested using Fisher's exact test and Pearson's *χ* ^2^ test for the categorical parameters and ANOVA for continuous parameters. Independent-samples analyzed by Mann-Whitney *U* test. All comparisons of the clinical scores between the patient and healthy control groups were performed by nonparametric tests. The neuropsychological tests were considered failed when the results were outside the normal range (means ±2 SDs obtained by the healthy control group).

## 3. Results

181 patients were screened through the database of the MS outpatient unit between years 2010 and 2011. A total of 61 consecutive patients who met the inclusion criteria were invited to participate in the study. Six patients refused to participate in the study. Nine patients with a Hamilton Depression Status Scale score 16 or above were excluded from the study. A series of 40 volunteers who had no history of neurological or psychiatric disease, alcoholism, or substance abuse were recruited from relatives of patients and community of neurology department.

Demographic and clinical characteristics of the patients and the healthy controls are presented in Table 1. There were no significant differences in age or education levels between CIS/MS and healthy control groups.

Table 2 displays the performance of patients and controls whose test was failed and their raw performances.

The highest performance of early CIS/RRMS group whose scores were outside the normal range that occurred in the following tests: TMT part B (26.1%), Stroop test (19.6%), RAVLT 1 (19.6%), RAVLT 2 (17.4%), TMT part A (17.4%), and phonological verbal fluency (15.9%).

Statistically significant differences were observed between patients and controls in the following tests: TMT part A (*P* = 0.03), TMT part B (*P* = 0.01), phonological verbal fluency (*P* = 0.01), and Stroop test (*P* = 0.01).

Of the 40 healthy controls 77% obtained scores outside the normal range in at least one test, 12.5% failed at least 2 tests, and 5.3% failed at 3 tests. Nobody in the control group failed at more than 4 tests. Therefore failure at 4 and more tests was considered as cognitive impairment. In early MS group 54.4% of subjects were impaired on a single cognitive measure, 4.3% were impaired on 2, and 20% were impaired on 3 cognitive measure. Based on the criteria employed 19.6% of early CIS/MS group (*N* = 9) screened positive for cognitive impairment (*P* = 0.003).

No significant relationship was found between cognitive impairment and age, sex, fatigue, and EDSS scores.

## 4. Discussion

Cognitive impairment should be considered among the main manifestations of MS even in the earliest stages of the disease. These deficits are a significant source of patient morbidity, impacting heavily on employment status and social functioning. Detecting such dysfunction as early as possible could help in adapting disease modifying therapeutic strategies. In the studies evaluating long term outcome of MS patients it is shown that cognitive impairment is one of the major reasons of neurological disability [8, 14]. Another problem is the identification of a brief and cost-effective instrument in order to screen subjects who deserve assessment through a comprehensive neuropsychological examination and specific interventions [15].

The nature of neuropsychological tests most frequently failed by early MS group suggested impairments focused on cognitive abilities such as attentional systems, cognitive flexibility, verbal fluency, executive functions, and learning, whereas semantic verbal fluency and visuospatial functions seemed to be spared. Although both fluency tasks draw on frontal and temporal lobe processes, the retrieval by letter is thought to be more dependent upon frontal lobe than on temporal lobe functioning, while retrieval by category is thought to be more dependent upon temporal as compared to frontal processes [16]. Barak et al. argue that CDT appears to be an effective and easily administered screen for early cognitive impairment in MS and report a sensitivity of 93.4% and a specificity of 85.8% in discriminating cognitively intact individuals with MS from those with cognitive impairment [11, 17]. We could not find a difference between CDT scores of early MS patients and healthy controls. Long term memory impairments are frequently reported in MS. Recent studies have shown that the primary memory problem is in the initial learning of information. Patients with MS require more repetitions of information to reach a predetermined learning criterion [18]. In our study 19.6% of the patients have impairment on RAVLT 1 which interpret learning and 17.4% of them have impairment on RAVLT-2 which interpret long term memory. But the results were not significant when compared with control group. Deficits of working memory and information processing speed which are interpreted by tests like PASAT, FST, and symbol digit modalities test (SDMT) are the most common cognitive deficits reported in MS [18]. PASAT tends to be associated with significant patient anxiety and on the contrary of other reports, PASAT seemed not to be appropriate to screen cognitive impairment in our patient group. Stroop test and TMT which have test durations of about 5 minutes can be used as screening tools for cognitive deficits.

Studies reporting on the frequency of disturbance of cognitive functions in MS patients show significant interpatient variability and depend on the methods used and on the type of patients examined [1]. According to our criterion (failure at 4 and more tests) 19.6% of early CIS/MS group showed cognitive impairment. The high number of failed tests in healthy control group may have been due to cut off values (2 SD). Other authors reported higher frequency of cognitive impairment which varied between 20% and 54% in early MS and CIS groups [1–7]. Most of them have lower cut off values and they required one or two failed tests to qualify the subjects as having cognitive impairment.

Fatigue has a high prevalence in MS and potentially makes worse cognitive deficit just as presence of anxiety or depression. But we could not find any association between fatigue scores and cognition. If the patient presents with anxiety or depression, a specific psychiatric interview should be performed before assessing cognition [18, 19].

It is now recognized that axonal injury due to inflammation and neurodegenerative processes already occur in the earliest stages of MS [3, 20]. The process of axonal loss, known to occur early in the disease process, might be associated with the current abnormal cognitive findings [1]. Thus, neuropsychological evaluation should be accomplished in the earliest stages of the disease even if neurological impairment is minimal.

## Figures and Tables

**Table 1:** Clinical and demographic characteristic of the patients and controls.

	Early MS	Controls
Number	46	40
CIS	8	—
RRMS	38	—
Age (mean, SD, range)	30.41 ± 9.36 (18–49)	34.7 ± 7.92 (22–50)
Gender (female/male)	30/16	26/14
Education years (mean, SD, range)	11 ± 3.85 (5–16 years)	11.4 ± 3.58 (5–16 years)
EDSS (median, range)	0.0 (0.0–5.0)	—
Disease duration at the time of evaluation, years (mean, SD, range)	1.11 ± 0.55 (2 months–2 years)	—
Time from onset of symptoms to definite diagnosis of RRMS, months (mean, SD, range)	5.97 ± 5.1 (1–24 months)	—

**Table 2:** Neuropsychological tests used and raw performances for healthy controls and patients. Results are expressed as percentages of controls and patients whose scores were outside the normal range for each cognitive task.

	Control (*N* = 40)		Early CIS/RRMS (*N* = 46)	
	Mean ± SD (range)	%	Mean ± SD (range)	%
PASAT 3 (3 sec correct responses)	41.63 ± 11.87 (24–60)	0	39.24 ± 13.32 (17–58)	4.3
PASAT 2 (2 sec correct responses)	33.55 ± 9.06 (21–55)	0	29.91 ± 9.88 (14–53)	10.9
FST (complete time/number of correct responses)	3.02 ± 0.79 (1.70–4.54)	8.7	3.06 ± 1.06 (1.71–6.12)	8.7
Phonological fluency (number of words in 60 sec)	39.29 ± 9.42 (21–62)	0	33.87 ± 13.27^*^ (13–65)	15.2
Semantic fluency (names of animals in 60 sec)	20.45 ± 4.00 (14–31)	0	19.78 ± 4.69 (12–32)	2.2
Stroop 1 (name the color print of color words, sec)	22.70 ± 4.58 (15–35)	0	25.11 ± 8.70^*^ (13–60)	19.6
Stroop 2 (name the color print of color words, errors)	0.75 ± 0.87 (0–3)	2.5	0.61 ± 0.74 (0–2)	0
TMT-A (complete time in sec)	40.75 ± 12.49 (22–70)	2.5	47.30 ± 16.85^*^ (16–90)	17.4
TMT-B (complete time in sec)	82.78 ± 28.10 (43–157)	5	103.48 ± 54.29^**^ (34–300)	26.1
RAVLT 1 (sum of number of words recalled after each repeat)	125.32 ± 12.03 (90–145)	5	116.61 ± 17.79 (77–140)	19.6
RAVLT 2 (total number of words recalled after 40-minute delay)	13.35 ± 1.25 (10–15)	5	12.26 ± 2.30 (5–15)	17.4
Clock drawing test	1.0	0	1.07 ± 0.32 (1–3)	4.4

^*^ *P* < 0.05.

^**^ *P* < 0.01.
